# Gastrointestinal Basidiobolomycosis with Biliary Tract Involvement in the Early Postpartum Period: A Case Report and Literature Review

**DOI:** 10.3390/idr18050102

**Published:** 2026-09-15

**Authors:** Abdullah Mohammed Alshehri, Abdulaziz Hassan Alamri, Khaled Abdulwahab Amer, Anas Khalid Alqarni

**Affiliations:** 1 Department of Gastroenterology, Aseer Central Hospital, Abha 62523, Saudi Arabia; drabdullahshehri@yahoo.com (A.M.A.); abdulazizhga@gmail.com (A.H.A.); 2 Department of Internal Medicine, Aseer Central Hospital, Abha 62523, Saudi Arabia; anasqarni@gmail.com

**Keywords:** *Basidiobolus ranarum*, gastrointestinal basidiobolomycosis, biliary tract, invasive fungal infection, postpartum, Splendore–Hoeppli phenomenon, Saudi Arabia, case report

## Abstract

**Introduction:** Gastrointestinal basidiobolomycosis is an uncommon invasive fungal infection caused by *Basidiobolus ranarum*, an environmental mould endemic to the arid south-west of Saudi Arabia and to a handful of other hot, dry regions. Its non-specific presentation routinely invites misdiagnosis as inflammatory bowel disease, tuberculosis or malignancy, and extension to the biliary tree is decidedly rare. **Case Presentation:** We report a 25-year-old Saudi woman who developed chronic watery diarrhoea and pronounced weight loss two months after her first delivery. Severe microcytic anaemia, leukocytosis, a very high erythrocyte sedimentation rate and multisegmental colonic wall thickening were initially attributed to inflammatory bowel disease complicated by *Clostridioides difficile* colitis. She subsequently required cholecystectomy for acalculous cholecystitis and then deteriorated, with gastric outlet obstruction and collections at the gallbladder bed. Re-examination of the gallbladder specimen revealed broad, pauci-septate hyphae enveloped by the Splendore–Hoeppli phenomenon, and culture yielded an isolate phenotypically identified as *B. ranarum*. Voriconazole followed by itraconazole achieved near-complete clinical and radiological recovery. **Discussion:** We hypothesise that the early puerperium could represent a possible, though as yet unproven, window of susceptibility to this infection. **Conclusions:** In endemic regions, gastrointestinal basidiobolomycosis should be considered when presumed inflammatory bowel disease behaves atypically; deep tissue sampling is often decisive, and prolonged triazole therapy can achieve an excellent outcome.

## 1. Introduction

*Basidiobolus ranarum* is a filamentous environmental fungus of the order Entomophthorales that is recovered from soil, decaying vegetation and the gut of amphibians, reptiles and insectivorous animals. Human disease was first documented in 1956 as a subcutaneous infection in Indonesia [1], and for several decades the organism was regarded almost exclusively as a cause of chronic subcutaneous mycosis in the tropics. The first gastrointestinal case followed in 1964 [2], and the gastrointestinal form—now termed gastrointestinal basidiobolomycosis (GIB)—has since become recognised as a distinct, if rare, clinical entity.

Reported cases cluster conspicuously in three arid corridors: the south-western provinces of Saudi Arabia, the south-western United States (chiefly Arizona) and southern Iran [3,4]. In these settings, warm temperatures, low rainfall and an abundant reptile population appear to favour environmental persistence of the fungus. Wall lizards native to the Saudi highlands carry *B. ranarum* in their intestinal tract, lending weight to an environmental or zoonotic route of acquisition through contaminated soil, water or food [5,6]. Sporadic cases have nonetheless been described well beyond these zones, including the central region of Saudi Arabia [7].

Clinically, GIB is a notorious mimic. It typically produces indolent abdominal pain, weight loss and a palpable mass that are readily mistaken for Crohn disease, intestinal tuberculosis or carcinoma [8,9,10]. Diagnosis is further hindered by the predominantly submucosal location of the organism, so that conventional endoscopic biopsies—which sample only the mucosa—are frequently unrevealing [6]. Spread beyond the bowel wall to the liver, retroperitoneum and, exceptionally, the biliary tree has occasionally been reported [11,12]. Here we describe a young woman who developed multifocal GIB with biliary tract involvement in the early postpartum period, and we use the case to frame a practical review of the diagnostic and therapeutic challenges that this infection poses in endemic regions.

## 2. Case Presentation

### 2.1. Patient Information and Clinical Findings

A 25-year-old Saudi woman was referred to Aseer Central Hospital, a tertiary centre in the highlands of south-western Saudi Arabia, for evaluation of three months of diarrhoea together with colonic wall thickening seen on an outside computed tomography (CT) scan. Her illness had begun roughly two months after the uncomplicated vaginal delivery of her first child. The earliest symptom was mild left lower-quadrant discomfort, which settled after she was treated empirically for *Helicobacter pylori* infection at a private clinic. Over the following weeks she developed watery, non-bloody diarrhoea about twice daily that occurred both day and night, bore no relation to meals, and was accompanied by an involuntary loss of 10–12 kg. She reported no fever, vomiting, rectal bleeding or night sweats.

Her medical, surgical and drug histories were unremarkable, and she had no known allergies. She was a lifelong non-smoker who kept house and had no recognised tuberculosis contact. There was a family history of *H. pylori* infection but none of inflammatory bowel disease (IBD), coeliac disease or gastrointestinal cancer. On examination she looked chronically unwell but was alert and haemodynamically stable. The abdomen was soft, non-tender and free of palpable mass, organomegaly, jaundice or peripheral lymphadenopathy.

### 2.2. Initial Investigations

Initial laboratory testing disclosed a constellation of abnormalities (Table 1): severe microcytic, hypochromic anaemia (haemoglobin 6.7 g/dL), neutrophilic leukocytosis (17.9 × 10^3^/µL), reactive thrombocytosis (602 × 10^3^/µL), a markedly raised erythrocyte sedimentation rate (ESR, 120 mm/h) and hypoalbuminaemia (2.75 g/dL). A stool nucleic-acid amplification test (PCR/NAAT) for toxigenic *Clostridioides difficile* was positive; glutamate dehydrogenase antigen and toxin A/B enzyme-immunoassay results are not documented in the available records. Tuberculin skin testing and mycobacterial PCR were negative, while a mildly elevated anti-tissue-transglutaminase IgA raised the possibility of coexisting coeliac disease. The differential white-cell count performed during the initial admission showed marked peripheral eosinophilia, with an absolute eosinophil count of 2.62 × 10^3^/µL (21.7% of a total leukocyte count of 12.07 × 10^3^/µL in that sample), and the peripheral blood smear confirmed moderate eosinophilia without eosinophil atypia—a finding of particular interest given the recognised association of basidiobolomycosis with peripheral eosinophilia. In view of the marked eosinophilia, stool examinations for ova and parasites were also performed and were negative.

**Figure 1:**
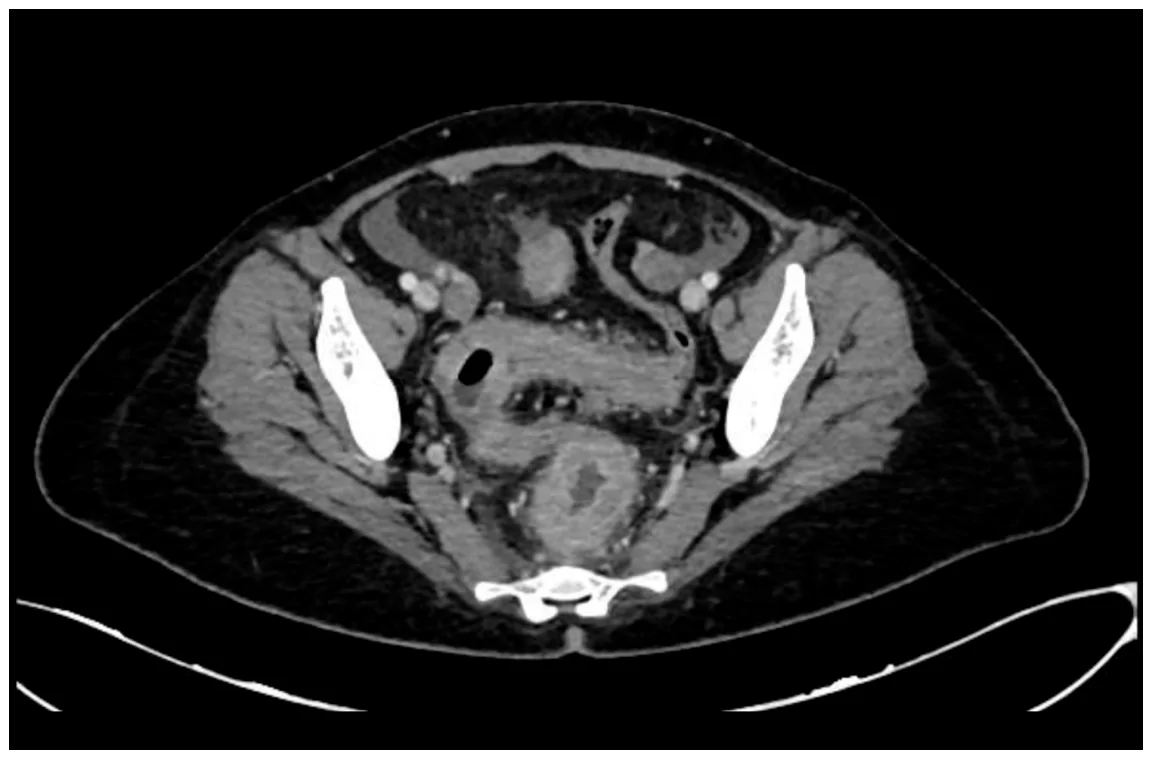
Initial contrast-enhanced CT of the abdomen and pelvis (axial). There is circumferential, multisegmental mural thickening of the large bowel with adjacent inflammatory fat stranding, an appearance initially attributed to inflammatory bowel disease.

**Figure 2:**
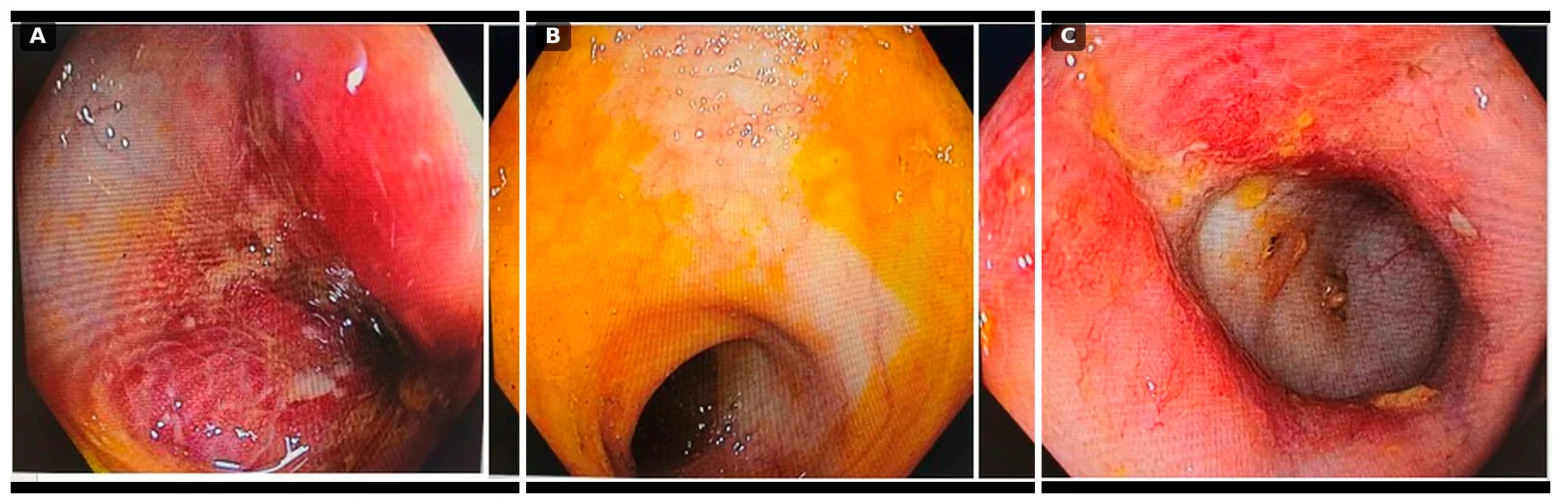
Initial colonoscopy. (**A**) Sigmoid colon with mucosal erythema, oedema and a shallow ulcer; (**B**) descending colon coated with yellowish exudate; (**C**) a more severely inflamed segment with luminal narrowing and contact friability.

Contrast-enhanced CT of the abdomen confirmed circumferential, multisegmental mural thickening of the large bowel with surrounding fat stranding, a pattern most in keeping with IBD (Figure 1). Colonoscopy showed patchy mucosal erythema, oedema, friability and shallow ulceration across several colonic segments; histology of the mucosal biopsies returned chronic active colitis without granulomas or malignancy, interpreted as compatible with either IBD or an infective colitis (Figure 2).

### 2.3. Hospital Course and Evolution

The patient received two units of packed red cells, which raised her haemoglobin to 9.7 g/dL, together with a course of oral vancomycin (125 mg four times daily) for *C. difficile* colitis. Her diarrhoea resolved completely and she was discharged with a plan for outpatient re-endoscopy.

**Figure 3:**
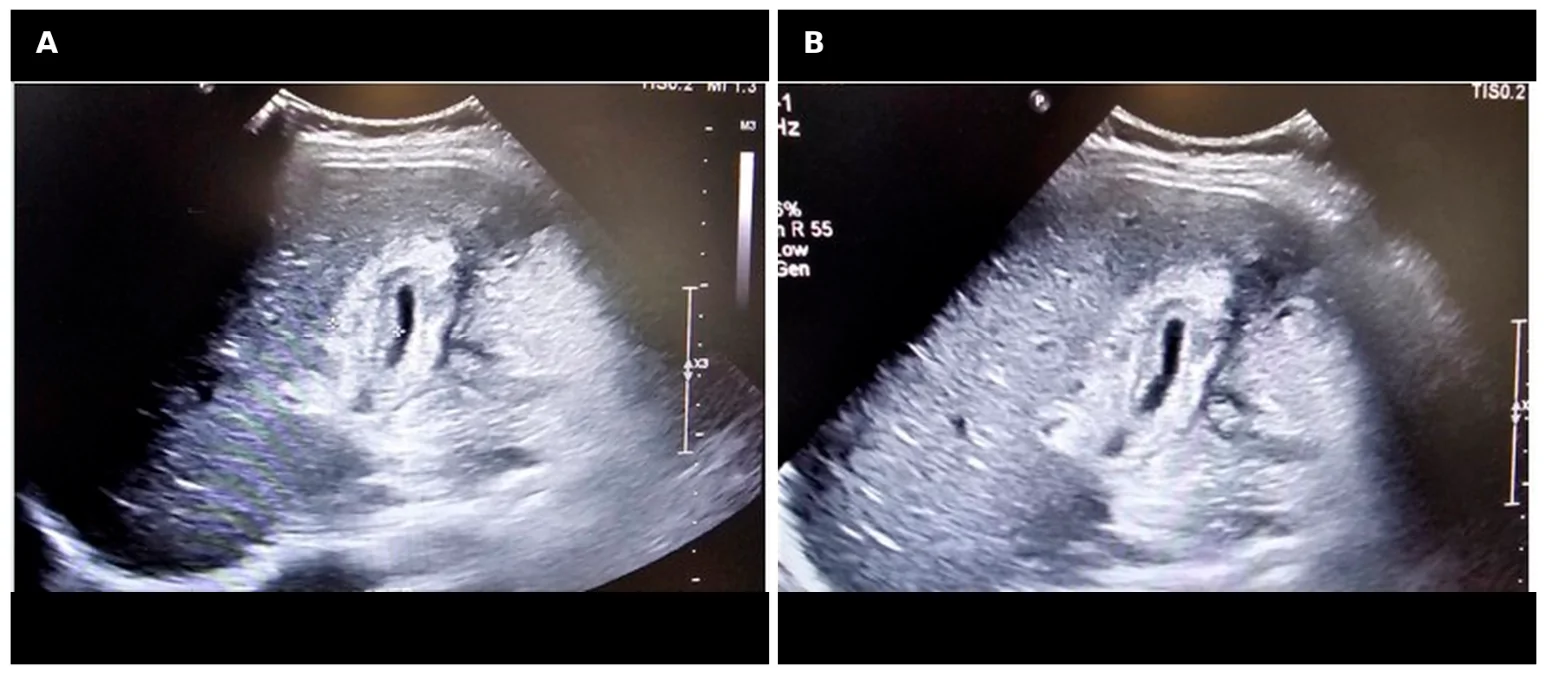
Abdominal ultrasonography of the gallbladder in two planes (**A**,**B**). The gallbladder is contracted with marked, diffuse wall thickening measured at 1.87 cm (callipers) and surrounding inflammatory change; no calculi are seen.

She returned about one month later with severe right upper-quadrant pain, nausea and vomiting. Ultrasonography demonstrated a contracted, markedly thick-walled gallbladder (wall thickness 1.87 cm) surrounded by inflammatory change but without calculi (Figure 3). She underwent cholecystectomy at an external facility. According to the operative record, the gallbladder was densely adherent to the stomach, colon and omentum, contained neither bile, pus nor stones, and was described by the surgeon as a “white solid structure”; histopathology reported active chronic inflammation with fibrosis and suppurative, epithelioid granulomas.

**Figure 4:**
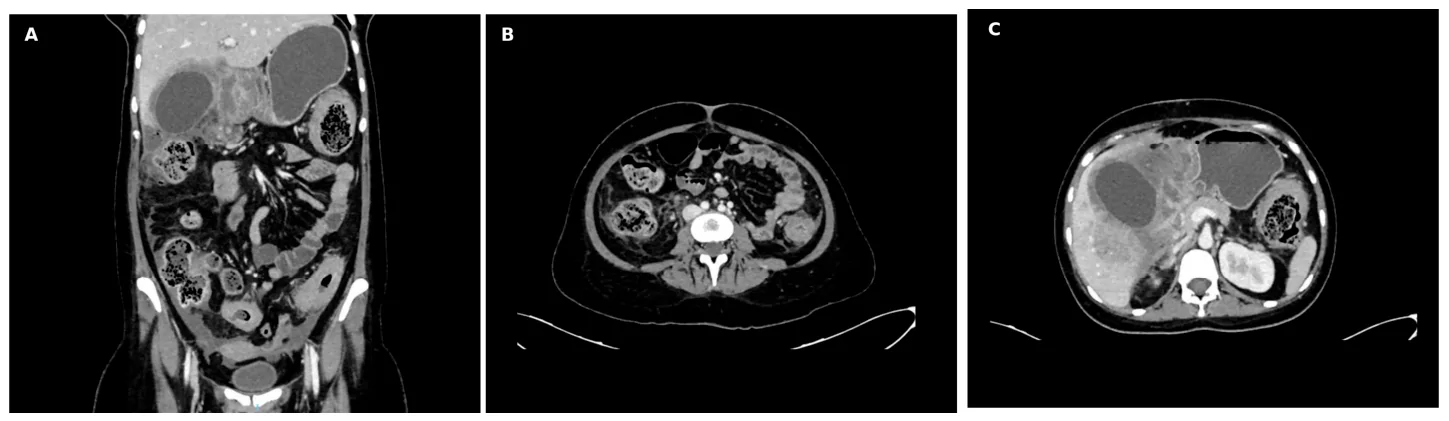
Repeat CT after cholecystectomy. (**A**) Coronal reformat showing a loculated collection in the gallbladder fossa and a distended, fluid-filled stomach reflecting gastric outlet obstruction; (**B**) axial image showing persistent multisegmental colonic wall thickening; (**C**) axial image of the upper abdomen showing the loculated subhepatic collection at the gallbladder bed.

**Figure 5:**
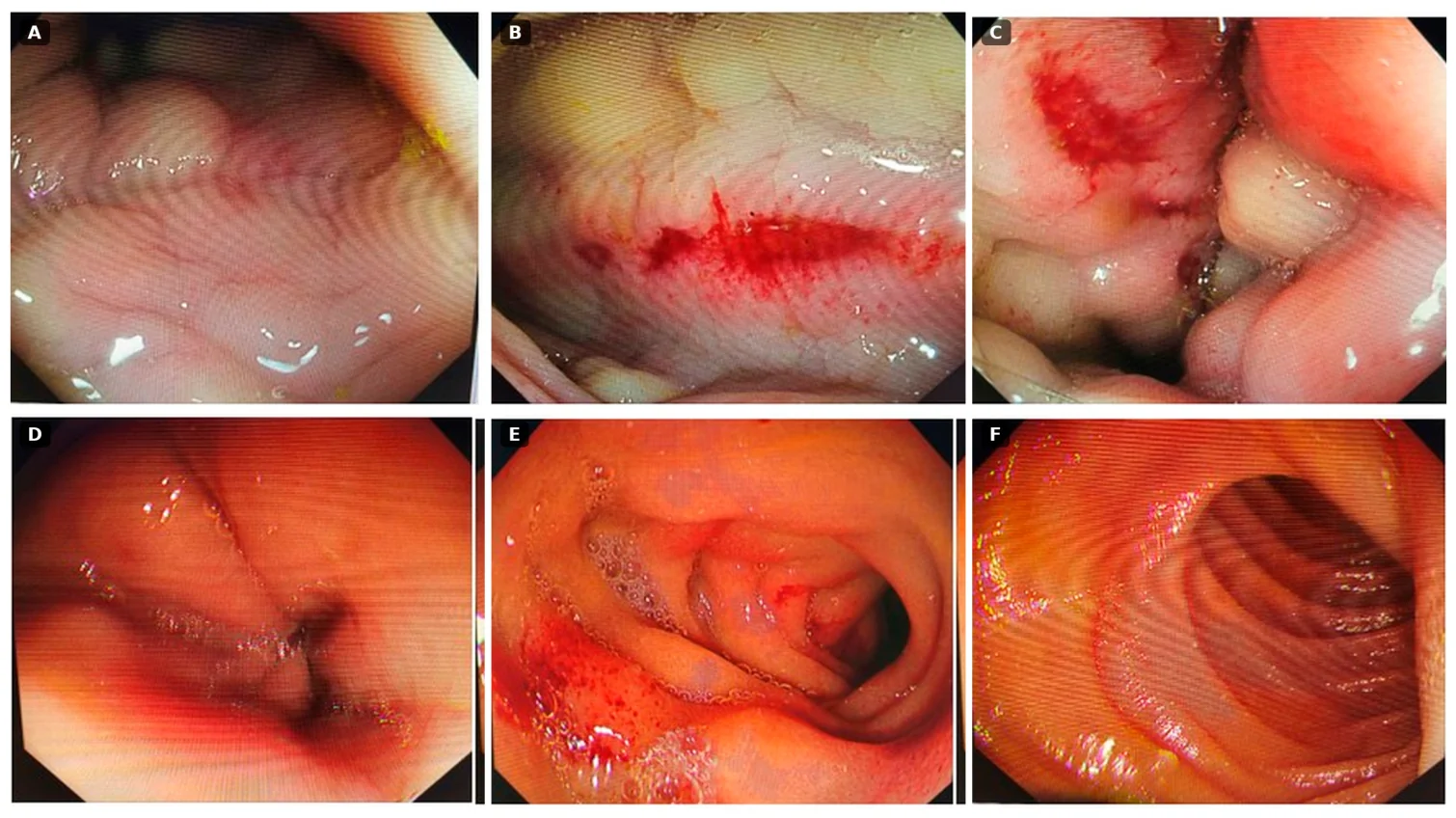
Upper gastrointestinal endoscopy. (**A**–**C**) Gastric antrum and body with mucosal oedema, erythema, nodularity, an area of active bleeding and superficial ulceration; (**D**–**F**) duodenal involvement with mucosal irregularity, friability and narrowing.

Rather than improving, her pain intensified within a week of surgery and she was readmitted to our hospital. Repeat CT revealed loculated fluid collections in the gallbladder fossa, distension of the stomach consistent with gastric outlet obstruction, and persistent multisegmental bowel-wall thickening (Figure 4). Percutaneous pigtail drainage of the collection yielded thick yellow-green pus. Upper endoscopy showed florid inflammation of the gastric antrum and duodenum, with mucosal oedema, erythema, nodularity, contact bleeding and superficial ulceration (Figure 5).

**Figure 6:**
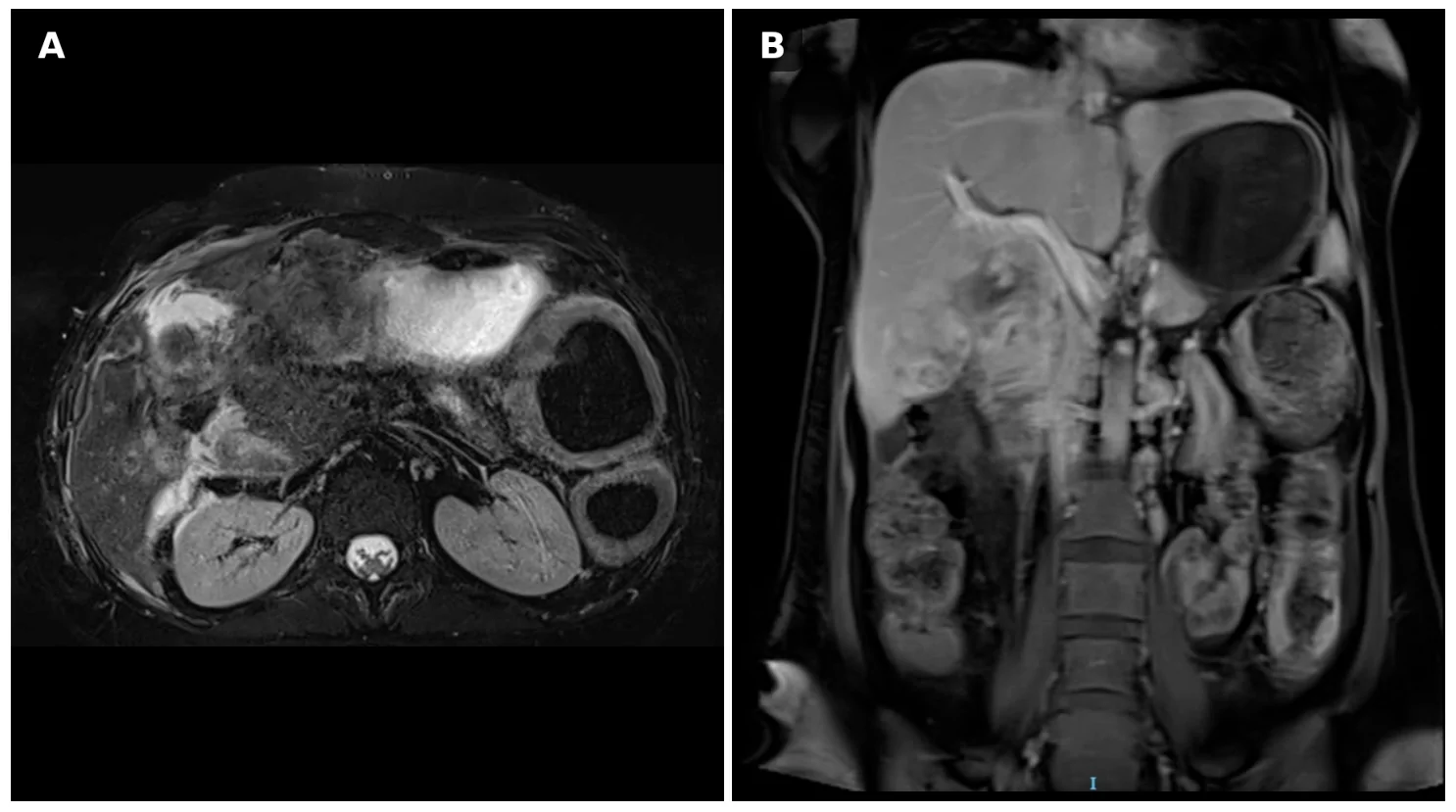
Contrast-enhanced MRI of the abdomen. (**A**) Axial image showing persistent inflammatory change at the gallbladder bed after cholecystectomy, extending into the adjacent hepatic parenchyma and the thickened gastric antrum; (**B**) coronal image showing periportal extension, skip-pattern bowel-wall thickening and a focal right renal lesion.

Contrast-enhanced magnetic resonance imaging (MRI) of the abdomen demonstrated circumferential thickening of the gastric antrum containing submucosal microabscesses, a skip-pattern of small- and large-bowel involvement, inflammatory infiltration of the gallbladder bed extending along the porta hepatis, and a focal lesion at the upper pole of the right kidney; the appearances were considered characteristic of an invasive fungal process, specifically basidiobolomycosis (Figure 6).

### 2.4. Diagnosis

**Figure 7:**
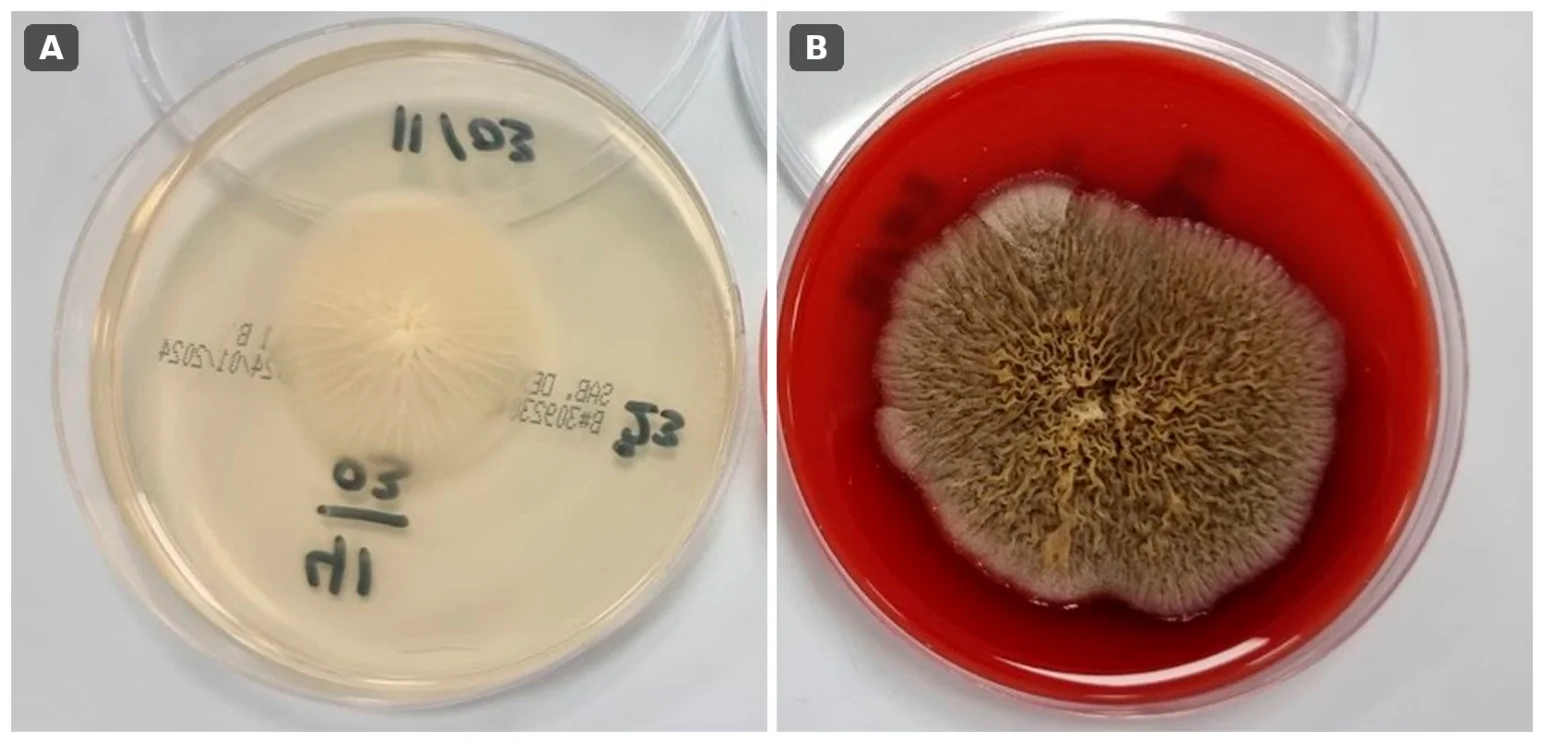
Mycological culture of the drained pus. (**A**) Sabouraud dextrose agar showing a waxy, glabrous, buff-coloured colony with characteristic radial folds; (**B**) blood agar showing the greyish-brown surface that develops on subculture—features consistent with *Basidiobolus ranarum*.

Prompted by the imaging, the cholecystectomy specimen was retrieved and re-examined with fungal stains. This disclosed broad, thin-walled, sparsely septate (pauci-septate) hyphae cuffed by intensely eosinophilic material—the Splendore–Hoeppli phenomenon—set within a dense eosinophilic and granulomatous infiltrate, findings that are highly characteristic of basidiobolomycosis. Culture of the drained pus on Sabouraud dextrose agar grew *Basidiobolus ranarum*, producing the expected waxy, glabrous, radially folded buff colonies that acquired a greyish-brown surface on subculture (Figure 7). Species-level identification was made by the clinical microbiology laboratory on the basis of these characteristic colonial and growth features, taken together with the histopathological demonstration of broad, pauci-septate hyphae with the Splendore–Hoeppli phenomenon; molecular confirmation (panfungal PCR with sequencing) was not available at our centre and could not be performed. A diagnosis of multifocal gastrointestinal basidiobolomycosis with biliary tract involvement was thereby established. Throughout this report, “biliary tract involvement” denotes histologically confirmed infection of the gallbladder with contiguous inflammatory extension along the gallbladder bed and porta hepatis, rather than demonstrated invasion of the bile ducts, and the multifocal distribution most plausibly reflects contiguous or intramural spread, as haematogenous dissemination was not microbiologically proven. Because the cholecystectomy and initial histopathological processing were performed at an external facility, photomicrographs of the stained sections were unfortunately not retrievable for publication.

### 2.5. Treatment and Outcome

**Figure 8:**
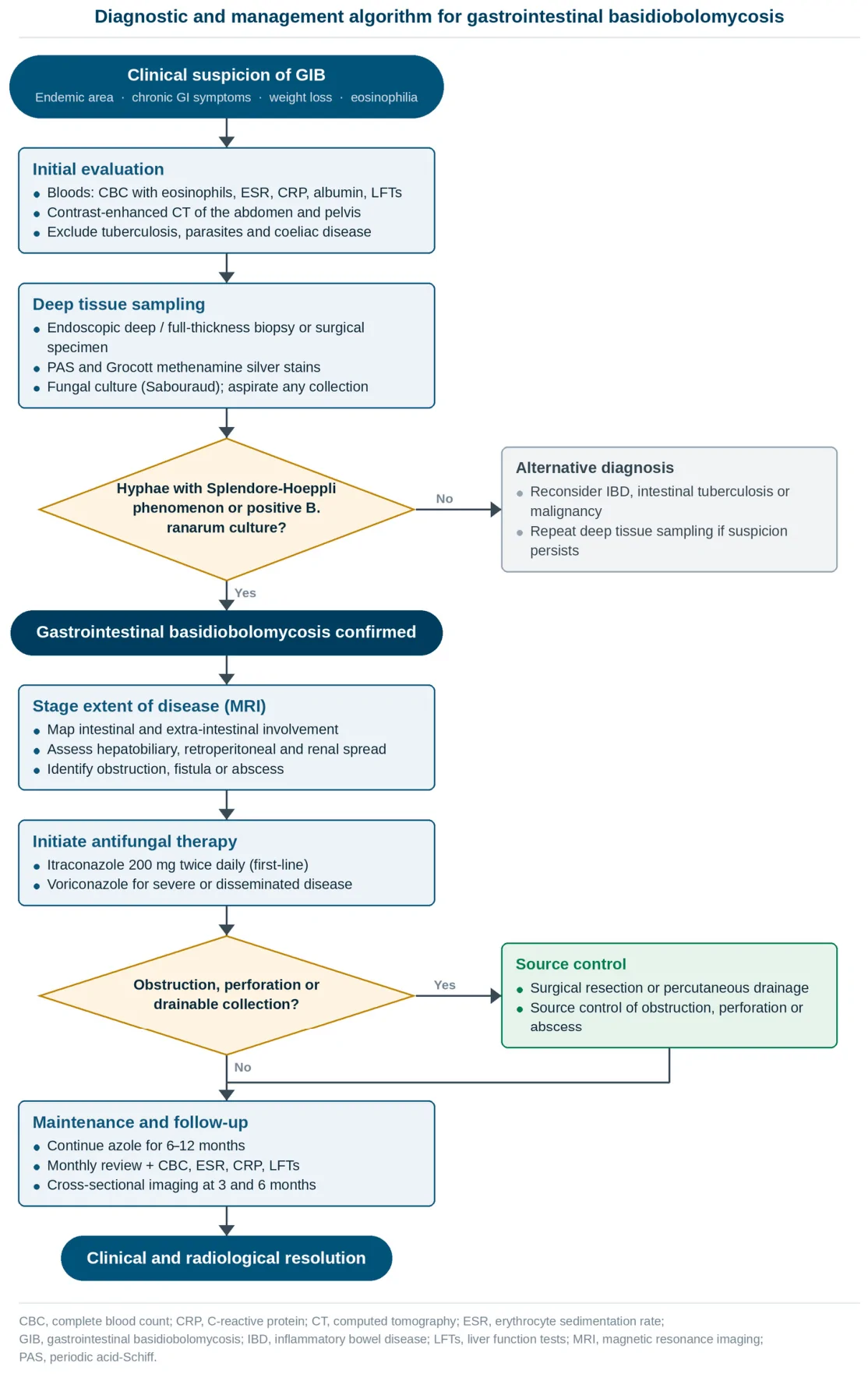
Proposed stepwise diagnostic and management algorithm for gastrointestinal basidiobolomycosis in endemic regions, emphasising early clinical suspicion, deep tissue sampling, mycological confirmation and prolonged triazole therapy with surgery reserved for complications. This pathway is a pragmatic synthesis of the available literature and the present case; it has not been prospectively validated and is not intended as a formal guideline.

Systemic antifungal therapy was started with intravenous voriconazole (a loading dose of 6 mg/kg every 12 h for one day, then 4 mg/kg every 12 h). Once she had stabilised, treatment was switched to oral itraconazole 200 mg twice daily for prolonged maintenance. Her symptoms abated steadily, the inflammatory markers normalised, and serial imaging showed near-complete resolution of the previously extensive disease. A practical synthesis of the diagnostic and management steps that this case illustrates is presented as an algorithm in the Discussion (Figure 8).

## 3. Discussion

For the accompanying narrative literature review, we searched PubMed/MEDLINE and Google Scholar from inception to June 2026 using combinations of the terms “basidiobolomycosis”, “*Basidiobolus ranarum*”, “gastrointestinal”, “hepatobiliary” and “biliary”, limited to English-language publications, and hand-searched the reference lists of retrieved articles for additional cases. This was a narrative rather than a systematic review; accordingly, the case counts and clinical frequencies cited below are approximate, are drawn from the referenced series and reviews, and may overlap between sources.

### 3.1. Epidemiology

Since the first gastrointestinal case in 1964 [2], GIB has been reported across several continents but remains heavily concentrated in a few hot, dry regions. In their landmark review, Vikram and colleagues assembled 44 cases worldwide and observed that roughly 43% originated in the United States—overwhelmingly Arizona—and about 25% in Saudi Arabia [3]. Within the Kingdom, the southern Aseer and Jazan provinces account for the great majority of reports (Table 2), a distribution that mirrors the local climate and the regional reptile reservoir of *B. ranarum* [5,13]. Recent Saudi series have reinforced this pattern and broadened the recognised spectrum, with contemporary bicentric and multicentre cohorts of children and adults documenting extensive colonic, hepatic and multifocal disease [14,15]. Our patient, from the Aseer highlands, fits the established geography of the disease.

### 3.2. Pathogenesis and Risk Factors

The precise route by which humans acquire GIB remains unproven, but ingestion of fungal elements in contaminated soil, water or food is the most widely accepted mechanism [5]. A distinctive and clinically important feature is that, unlike most invasive mycoses, GIB predominantly affects immunocompetent hosts. Recognised associations include residence in an endemic area, contact with soil or reptiles, and the use of acid-suppressing medication, which may weaken the gastric barrier to ingested spores. Pregnancy and the puerperium have occasionally featured among reported patients, and the temporal association in our case—symptoms beginning some two months postpartum—is noteworthy, although it may be coincidental. The physiological immune re-modelling of late pregnancy and the early postpartum period—characterised by a relative shift away from cell-mediated (T-helper-1) immunity—could plausibly open a transient window of vulnerability to an organism that immunocompetent hosts would ordinarily contain. We advance this as a hypothesis rather than a demonstrated causal link.

### 3.3. Clinical Presentation

The clinical hallmark of GIB is its lack of specificity. Abdominal pain, a palpable mass, altered bowel habit, weight loss and low-grade fever predominate, and these features overlap almost completely with Crohn disease, intestinal tuberculosis and colorectal carcinoma (Table 3) [8,9,10]. Peripheral eosinophilia, present in the large majority of cases, is among the few clues that should prompt consideration of a fungal cause. The colon—particularly the caecum and ascending colon—is the segment most often involved, although disease may arise anywhere from the stomach to the rectum; the prominent gastroduodenal involvement in our patient is a reminder of this range. Extension beyond the bowel to the liver, retroperitoneum and biliary tree is uncommon. Biliary tract involvement of the kind seen here—histologically confirmed gallbladder infection with contiguous peri-portal extension on imaging, rather than proven intraductal disease—has been described in only a small number of patients [11,12], and whether this reflects genuine rarity or simply under-recognition—given how difficult the diagnosis is to secure—remains unclear.

### 3.4. Diagnostic Approach

Three factors conspire to delay the diagnosis: the non-specific clinical picture, the submucosal habitat of the organism, and limited clinician familiarity with the disease [6,19]. Cross-sectional imaging usually shows circumferential bowel-wall thickening, an inflammatory mass or submucosal abscesses; MRI is especially valuable for mapping the extent of disease and for revealing the characteristic skip pattern, as it did in our patient [20]. The diagnosis ultimately rests on tissue. Histology characteristically shows chronic granulomatous inflammation, a dense eosinophilic infiltrate, and broad, pauci-septate hyphae surrounded by the Splendore–Hoeppli phenomenon [21]. Because the fungus lies deep to the mucosa, superficial endoscopic biopsies are frequently falsely negative, and—as in this case—the diagnosis may only declare itself on a surgical specimen. Culture supports mycological identification: *B. ranarum* characteristically grows on Sabouraud dextrose agar at 25–37 °C within two to three days, forming waxy, glabrous colonies with radial folds [17]. Where available, panfungal PCR and sequencing can provide definitive species-level confirmation and shorten the diagnostic interval.

### 3.5. Management

Treatment combines prolonged systemic antifungal therapy with surgery reserved for specific complications (Table 4). Triazoles are the mainstay: itraconazole is the most widely used first-line agent, with reported response rates of 70–80%, while voriconazole—used here—is an effective option for severe or disseminated disease [18,22,23]. Therapy is necessarily long, usually six to twelve months, and should continue until both symptoms and imaging have resolved. The place of surgery has narrowed over time. Although earlier reports relied heavily on resection, accumulating experience indicates that many patients can be managed medically once the diagnosis is secured, with operation reserved for obstruction, perforation, abscess or diagnostic uncertainty [22,24]; in children, mechanical complications such as intussusception have also mandated operative intervention [25]. Saturated potassium iodide solution, long used for subcutaneous basidiobolomycosis, is a further inexpensive and readily available option: it has produced complete resolution of gastrointestinal disease refractory to amphotericin B and itraconazole in at least one paediatric case, and it features in several of the combination regimens reported in recent series [24,26]. Statements about amphotericin B should be interpreted cautiously: in vitro susceptibility of *B. ranarum* is variable and clinical failures have been reported, but the evidence base rests on small numbers of heterogeneous cases. A recent qualitative review of 24 cases reaffirmed the central role of azoles and the variable, often adjunctive, contribution of surgery [24]. In our patient, source control of the drainable collection together with extended triazole therapy produced an excellent outcome without further bowel resection.

### 3.6. Key Learning Points

Three aspects of this case merit emphasis. First, the onset in the early puerperium raises the hypothesis—unproven, and possibly coincidental—that the immunological changes of pregnancy and the postpartum period might create a transient susceptibility to invasive fungal infection; this association is worth bearing in mind in endemic regions. Second, histologically documented biliary tract involvement, reported in only a handful of previous patients, extends the recognised anatomical reach of GIB. Third, the early course—dominated by *C. difficile* colitis and a mildly positive coeliac serology—illustrates how readily GIB is mistaken for commoner conditions, and how a deep tissue specimen, rather than a superficial biopsy, is often the key to the diagnosis. A pragmatic, stepwise approach to suspected GIB in endemic areas is summarised in Figure 8.

## 4. Conclusions

We describe an unusual case of multifocal gastrointestinal basidiobolomycosis with biliary tract involvement arising in the early postpartum period in a young woman from the Aseer region of Saudi Arabia. The case suggests three lessons: the puerperium may possibly represent a window of susceptibility to this infection, although this remains a hypothesis; biliary tract involvement should be considered when GIB extends beyond the bowel; and a high index of suspicion is indispensable in endemic settings. Because superficial biopsies so often miss the submucosal organism, early deep tissue sampling and the prompt initiation of triazole therapy are decisive in achieving a favourable outcome.

## Figures and Tables

**Table 1:** Laboratory findings at presentation.

Parameter	Value	Reference Range	Interpretation
Haemoglobin	6.7 g/dL	12–16	Severe anaemia
White cell count	17.9 × 10^3^/µL	4–11	Leukocytosis
Eosinophils	2.62 × 10^3^/µL (21.7%) ^a^	0.02–0.5	Marked eosinophilia
Platelets	602 × 10^3^/µL	150–400	Thrombocytosis
MCV	60 fL	80–100	Microcytic
MCH	17.4 pg	27–31	Hypochromic
RDW	20.6%	11.5–14.5	Anisocytosis
ESR	120 mm/h	0–20	Markedly elevated
Albumin	2.75 g/dL	3.5–5.0	Hypoalbuminaemia
Anti-tTG IgA	27.1 U/mL	<20	Mildly elevated
*C. difficile* PCR (NAAT)	Positive	Negative	Toxigenic *C. difficile* detected; treated as *C. difficile* infection
Stool ova and parasites	Negative	Negative	No parasitic infection detected

ESR, erythrocyte sedimentation rate; MCH, mean corpuscular haemoglobin; MCV, mean corpuscular volume; NAAT, nucleic-acid amplification test; PCR, polymerase chain reaction; RDW, red cell distribution width; tTG, tissue transglutaminase. ^a^ Differential leukocyte count and peripheral blood smear were performed during the initial admission on a separate blood sample from the complete blood count shown (total leukocyte count 12.07 × 10^3^/µL).

**Table 2:** Geographic distribution of reported cases of gastrointestinal basidiobolomycosis.

Region	Approximate Cases	Representative References
South-western Saudi Arabia (Aseer, Jazan)	>60	[3,14,15,16]
United States (Arizona)	17–19	[3,4]
Southern Iran	>25	[11,17]
Kuwait and Iraq	8–10	[18]
Other regions	Sporadic	[7,10]

Case counts are approximate and drawn from the cited reviews and series; overlap between sources is likely.

**Table 3:** Frequency of clinical features of gastrointestinal basidiobolomycosis reported in the literature.

Clinical Feature	Reported Frequency	Comment
Abdominal pain	85–94%	Most common symptom
Abdominal mass	30–78%	May mimic malignancy
Constipation	70–83%	Common in children
Weight loss	50–60%	Often substantial
Fever	22–40%	Variable; may be absent
Peripheral eosinophilia	80–94%	Key diagnostic clue

Frequencies are pooled approximations from the cited reviews and case series [3,8,9,10].

**Table 4:** Antifungal agents used in the treatment of gastrointestinal basidiobolomycosis.

Agent	Route	Reported Efficacy	Notes
Itraconazole	Oral	70–80%	First-line; typically 6–12 months
Voriconazole	IV or oral	Effective	Severe or disseminated disease
Posaconazole	Oral	Effective	Limited published experience
Amphotericin B	IV	Variable; failures reported	In vitro resistance and clinical failures reported in limited case data; interpret cautiously
Potassium iodide (saturated solution)	Oral	Reported cures in case reports	Inexpensive adjunct or salvage option; response reported in azole/amphotericin-refractory disease [26]

IV, intravenous. Efficacy estimates are derived from heterogeneous case reports and small series [17,18,22,23,24,26].

## Data Availability

The data supporting the findings of this case report are contained within the article; further detail is available from the corresponding author upon reasonable request.

## Abbreviations

The following abbreviations are used in this manuscript:

CT	computed tomography
ESR	erythrocyte sedimentation rate
GIB	gastrointestinal basidiobolomycosis
GMS	Grocott methenamine silver
IBD	inflammatory bowel disease
MRI	magnetic resonance imaging
PAS	periodic acid–Schiff
PCR	polymerase chain reaction
